# Association between Bitter Taste Sensitivity and Weight Status in Adults: A Systematic Review and Meta-Analysis

**DOI:** 10.1093/nutrit/nuaf160

**Published:** 2025-08-28

**Authors:** Alia Shareef, Getahun Fentaw Mulaw, Kruti Rathore, Chris Irwin, Lisa Vincze, Roshan Rigby, Rati Jani

**Affiliations:** School of Allied Health, Sport and Social Work, Griffith University, Gold Coast, Queensland 4222, Australia; School of Pharmacy and Medical Science, Griffith University, Gold Coast, Queensland 4222, Australia; Department of Public Health, College of Health Sciences, Woldia University, Woldia 6040, Ethiopia; Department of Nutrition and Dietetics, Bangalore University, Bengaluru 560056, India; School of Allied Health, Sport and Social Work, Griffith University, Gold Coast, Queensland 4222, Australia; School of Allied Health, Sport and Social Work, Griffith University, Gold Coast, Queensland 4222, Australia; Faculty of Health Sciences and Medicine, Bond University, Gold Coast, Queensland 4229, Australia; School of Allied Health, Sport and Social Work, Griffith University, Gold Coast, Queensland 4222, Australia

**Keywords:** bitter taste sensitivity, weight status, TAS2Rs, meta-analysis, diet

## Abstract

**Context:**

Bitter taste sensitivity plays a role in dietary preferences and food intake, potentially affecting weight status. Despite previous studies performed to investigate this role, the relationship between bitter taste sensitivity and anthropometric measures remains inconclusive.

**Objective:**

In this review we sought to systematically evaluate the association between sensitivity to bitter taste and weight status in adults through a comprehensive meta-analysis and meta-regression.

**Data Sources:**

We conducted a systematic search of the EMBASE, MEDLINE, CINAHL, Scopus, and PsycInfo databases for reported studies on this topic published up to March 2024. Included studies involved healthy adults and were published in English.

**Data Extraction:**

Two reviewers independently screened titles, abstracts, and full texts. Data on study characteristics, methods of assessing taste sensitivity, and anthropometric outcomes were extracted. Quality assessment was performed using the American Dietetic Association Quality Criteria Checklist.

**Data Analysis:**

Meta-analysis of 27 studies (80 effect estimates) revealed no significant association between bitter taste sensitivity and overall weight status (Cohen’s *dz*, −0.02; 95% CI, −0.10 to 0.05, *P = *.58). Subgroup analyses also showed no significant differences between nontasters, medium tasters, and supertasters. Meta-regression indicated that the waist–hip ratio was a significant moderator (*P = *.004). Heterogeneity was high (*I*^2^ = 91.5%), and publication bias was minimal.

**Conclusions:**

Bitter taste sensitivity does not significantly influence overall weight status but may affect body fat distribution. Methodological variability and population differences highlight the need for standardized protocols in future research. These findings contribute to understanding taste sensitivity in obesity prevention and dietary behavior strategies.

## INTRODUCTION

Globally, more than 1.9 billion adults are classified as being overweight, and 650 million are obese.^1^ Several studies have demonstrated a clear inverse relationship between obesity and health-related quality of life, as well as life expectancy.^2^ It is also well established that obesity increases susceptibility to developing several chronic diseases, including type 2 diabetes, stroke, coronary artery disease, and numerous cancers.^3^ A previous meta-analysis involving 239 studies (with over 10 million participants) have highlighted a clear positive relationship between body mass index (BMI) and all-cause mortality; the hazard ratio (HR) increased from 1.11 for individuals with a BMI of 25.0-30 kg/m^2^ to 2.71 for those with a BMI of 40.0-60.0 kg/m^2^.^4^ At an economic level, obesity has a profound impact on healthcare costs, accounting for over 30% of direct healthcare costs and almost 70% of indirect costs.^5^ As such, increased understanding of factors contributing to obesity is essential for the development of effective prevention and management strategies.

Dietary intake is a major determinant and modifiable factor in obesity.^6^ However, the dietary behaviors of individuals are profoundly influenced by taste sensitivity, an area of nutritional research that has received increased attention over recent decades.^7–9^ Humans possess intricate sensory transduction pathways that facilitate the differentiation of at least 5 basic tastes—sweet, bitter, salty, sour, and umami—essential for assessing the quality and safety of food.^8^ In particular, the perception of bitterness serves as a protective mechanism to deter consumption of potentially toxic substances, although not all bitter-tasting foods are harmful.^10^

The genetic underpinnings of bitter taste sensitivity have been the subject of extensive investigation. Although humans possess approximately 25 taste receptor type 2 (TAS2R) functional bitter taste receptors, each responsive to different bitter compounds, research on individual variation in bitter taste perception has largely focused on TAS2R member 38 (TAS2R38).^11^ This receptor is activated by prototypical compounds such as phenylthiocarbamide (PTC) and propylthiouracil (PROP), and its common genetic variants—particularly the PAV (proline-alanine-valine) and AVI (alanine-valine-isoleucine) haplotypes—are associated with well-documented differences in perceived bitterness.^12^^,^^13^ TAS2R38 genotype detection is used to help classify individuals as nontasters, tasters, or supertasters, reflecting variable sensitivity to PROP/PTC. Although these phenotypic variations are strongly influenced by TAS2R38 polymorphisms, the polymorphisms are not entirely determined by these variations, suggesting additional genetic or environmental contributions.^14^^,^^15^ Due to the availability of both well-characterized ligands and genotyping methods, TAS2R38 has become a widely used model for studying bitter taste sensitivity.^16^^,^^17^ Heightened sensitivity to bitter compounds may deter individuals from consuming certain health-promoting bitter vegetables (eg, cruciferous varieties),^18^ potentially leading to lower dietary quality and a shift toward more palatable, energy-dense foods.^19^ This avoidance of vegetables may contribute to increased energy intake or imbalanced macronutrient consumption, thereby influencing weight status.^20^

Previous reviews investigating the relationship between bitter taste sensitivity and weight status have yielded conflicting results. Although some investigators have reported study results suggesting an inverse relationship between bitter taste sensitivity and weight status, others have reported a positive association or no association at all. For instance, the systematic review by Fathi et al. included primarily case-control studies, most of which were conducted in White populations.^21^ Of these, 7 studies found lower sensitivity to bitterness in overweight or obese individuals, while others reported no significant association. Similarly, the 2 narrative reviews revealed conflicting findings. Shizukuda et al.^22^ identified studies showing both positive and negative correlations between bitter taste sensitivity and obesity and others that found no association. In another review, Tepper et al.^23^ also reported mixed results, with some studies suggesting an inverse relationship between PROP tasting and calorie intake or BMI, while other studies presented opposing conclusions.

These discrepancies may stem from methodological variations, particularly in how bitter taste sensitivity is assessed. Differences in the compounds used (eg, PROP or quinine), testing protocols, and classification thresholds for taster categories can significantly influence study outcomes. Moreover, bitter taste sensitivity can be measured through distinct psychophysical constructs, each reflecting a different stage of perceptual processing.^24^ The detection threshold (DT) refers to the lowest concentration of a tastant that elicits a perceptible sensation, without requiring the participant to identify the taste quality.^24^^,^^25^ The recognition threshold (RT) is the concentration at which the specific taste quality—such as bitterness—can be correctly identified.^25^ In contrast, suprathreshold taste intensity (STT) methods assess how strong or intense the taste is perceived to be once it is clearly detectable. Scaling tools such as the general labelled magnitude scale (gLMS) are often used for these assessments.^25^^,^^26^ In addition, individual characteristics such as age, sex, genetic variation, and lifestyle factors—including diet and cultural context—can further influence outcomes.^27^^,^^28^ Collectively, these factors underscore the need for a comprehensive and systematic meta-analytical approach to clarify the relationship between bitter taste sensitivity and weight status.

Given the substantial heterogeneity in findings and the absence of a unified meta-analytical investigation, there is a clear need to systematically synthesize the evidence. In this review we aimed to quantify the strength of the association between bitter taste sensitivity and weight status through meta-analysis and meta-regression. Additionally, we also explored potential moderating factors. To our knowledge, no other study to date has systematically investigated these associations in adult populations using a meta-analytical approach. Therefore, this review was intended to fill an important gap in the literature and provide more robust evidence to guide future research and inform public health strategies concerning obesity and dietary behavior.

## METHODS

### Study Design and Search Strategy

This study was conducted according to the guidelines outlined in the Preferred Reporting Items for Systematic Reviews and Meta-Analyses (PRISMA) 2020.^29^ This review was pre-registered with PROSPERO (ID number CRD42024525085). A systematic literature search of 5 electronic scientific databases (EMBASE, MEDLINE, CINAHL, Scopus, and PsycInfo) was performed in March 2024, and the review was managed using Covidence software (https://www.covidence.org/). The search was limited to research involving humans, published in the English language. The research methodology was structured around the PICO (Population, Intervention, Comparator, Outcome) framework, as detailed in the supplementary materials (Table S2), which guided the formation of the research question. The search strategy included terms, phrases, and subject headings related to research question concepts (eg, genotype, phenotype, bitter, taste, weight, weight status, and adults). The systematic approach to the literature search, including the specific keywords applied, is presented in Table S3. To ensure a comprehensive search, we conducted a validation check by cross-referencing located systematic reviews, meta-analyses, and primary research articles.^29^^,^^30^ The selection process for relevant literature incorporated a tiered screening method with an initial review of titles and abstracts followed by a thorough evaluation of full-text articles.

### Eligibility Criteria

Studies were included in the review if they were conducted with generally healthy adult participants. Due to differences in definitions of adult age across several countries,^31^ we decided to include studies involving participants who were 17 years of age or older. No restrictions were placed on publication dates, type of study design, participant sex, or study location. Requirements for included studies were testing and measurement of taste sensitivity, intensity, thresholds, and acuity, and/or perception of bitter taste by either genotyping methods (eg, blood samples, saliva samples, cheek cell samples) or phenotyping methods using either STT, DT, or RT methodologies (eg, PTC/PROP taste strips, filter paper, solutions, and/or certain bitter food/beverages). Studies examining the association between bitter taste sensitivity (eg, genotype and/or phenotype) and adult weight status (eg, weight, BMI, waist circumference, waist–hip ratio, or body fat percentage) were eligible for inclusion in the review. For studies that categorized bitter taste sensitivity groups based on TAS2R38 genotype single nucleotide polymorphisms (SNPs), unless the author categorized them in the article, we identified them as follows: PAV/PAV = supertasters, PAV/AVI = medium tasters, and AVI/AVI = nontasters.^15^ For studies that examined the *TAS2R38* gene rs10246939 genetic marker, categories were determined as follows: CC = supertaster, CT = medium taster, and TT = nontaster.^32^

For studies reporting both phenotypic and genotypic data, both datasets were included in the meta-analysis.^33^ This decision was based on evidence that the TAS2R38 genotype only explains approximately 50%-65% of the variance in PROP bitterness intensity, supporting the nonredundancy of these 2 measures.^34^^,^^35^ The inclusion of both datasets preserved the full range of available information and maximized the statistical power.^36^ Studies were excluded if they encompassed participants with chronic diseases, developmental disabilities, autism spectrum disorder, other cognitive or intellectual impairments, or sensory-based feeding difficulties (eg, dysphagia). In addition, studies categorized as animal-based research, unpublished reports, dissertations, narrative/scoping/systematic/other reviews, letters to the editor, editorials, commentaries, or case studies were also excluded.

Studies were deemed eligible for inclusion in the meta-analysis if they included adequate data to compute effect sizes (ie, Cohen’s *dz*), along with associated measures of variance (ie, 95% CIs). Studies were also required to categorize participants into distinct tasting groups (ie, non-tasters, medium tasters, and/or supertasters). Genotype-based categorization relied on TAS2R38 polymorphisms (eg, PAV/PAV, PAV/AVI, AVI/AVI), which were mapped to phenotypic tasting groups based on established associations in prior literature. Studies that lacked sufficient data or did not categorize participants into taster status were considered for inclusion in the narrative synthesis only. In cases in which multiple studies shared the same participant pool and examined identical research inquiries, preference was given to the study with the largest sample size and/or the most comprehensive dataset. This approach was adopted to prevent duplication of data in the meta-analysis.

### Data Screening, Extraction, and Quality Assessment

Two researchers, A.S. and K.R., conducted individual reviews of abstracts to ensure adherence to predefined selection criteria. Discrepancies in their evaluations were adjudicated by a third reviewer (R.J., R.R., or L.V.). Full texts of articles identified through the initial abstract screening process were independently assessed for eligibility and comprehensiveness.

To gauge the level of agreement between the 2 initial reviewers, Cohen’s kappa coefficient (κ) was computed. This statistical measure provides insight into interrater agreement, with values of ≤0.00-0.20 = slight agreement, 0.21-0.40 = fair agreement, 0.41-0.60 = moderate agreement, 0.61-0.80 = substantial agreement, and 0.81-1.00 = almost perfect agreement.^37^ In this study, the kappa coefficient for interrater agreement was found to be 0.93.

The data extraction process involved gathering information on the following variables: year of publication; country in which the study was conducted; type of study (eg, cross-sectional, cohort, randomized controlled trial [RCT]); details of the genotype and/or phenotype related to taste sensitivity; methods used for bitter taste testing (eg, PROP or PTC testing); age, sex, and ethnicity of participants; total sample size; and weight status measurements (BMI, waist circumference, waist–hip ratio, body fat percentage). Data extraction was carried out independently by 2 researchers (A.S. and G.F.M.), with discrepancies resolved by a third researcher (R.J.).

The risk of bias and the methodological quality were assessed independently by K.R. and A.S. using the American Dietetic Association Quality Criteria Checklist (QCC) (https://www.andeal.org/evidence-analysis-manual). The QCC rates the quality of the study as positive, negative, or neutral, reflecting the risk of bias in participant selection, generalizability, data collection, and analysis. This tool was selected due to its broad applicability across diverse research designs and track record of yielding higher levels of interrater reliability than alternate instruments.^38^ Each study was scored in accordance with the following categories: positive = comprehensive treatment of critical methodological concerns such as inclusion and exclusion criteria, bias, generalizability, analytical rigor, and data collection methods; neutral = methodological robustness was neither markedly deficient nor notably outstanding; negative = failure to address these methodological concerns sufficiently. Studies found to have a negative rating for study quality were not excluded. In instances of disagreement between K.R. and A.S., a consultative process was initiated with a third reviewer (R.J., R.R., or L.V.) to achieve consensus.

### Statistical Analysis

In this comprehensive meta-analysis, study design was not a limiting factor, allowing for a wide array of effect estimates (eg, mean, *β*, *r*, and odds ratio) and measures of variability (eg, SD, 95% CIs, and SEs) to be included. The methodological diversity in the primary literature regarding the quantification of weight status was subsequently standardized into Cohen’s *dz* and its corresponding variance for uniformity and ease of comparison across studies.

Multilevel meta-analyses were performed using R Studio (release 2024.04.2 + 764) and the *“metafor”* package.^36^ Figures were developed using the *“ggplot2”* package.^39^ Three sources of variance were modeled in the analyses: level 1: sampling variance for the observed effect estimates; level 2: variance between effect estimates derived from the same studies; and level 3: variance between studies. Mean effect estimates were considered statistically significant when the 95% CI did not include zero. Heterogeneity was assessed using Cochran’s *Q*, the *I^2^* index, and the within- and between-cluster variance components (ie, *σ^2^*). Significant heterogeneity was indicated by *P *< .05 for Cochran’s *Q.*^40^

Subgroup analyses were conducted to compare weight-related outcomes (eg, BMI, waist circumference, waist–hip ratio, body fat percentage) between nontasters, medium-tasters, and supertasters. Cohen’s *dz* was used to standardize effect sizes across studies. A random-effects model was applied to account for variability, and heterogeneity was assessed using Cochran’s Q test and the *I^2^* statistic.

Funnel plots were assessed graphically, and the Egger’s regression test was examined for publication bias.^41^ Analyses were considered biased if the intercept of the regression test (including the SE of the effect sizes as a moderator) differed from zero at *P = *.10. Restricted maximum likelihood (REML) multilevel simple meta-regression analyses were performed to explore potential sources of heterogeneity and the influence of independent moderators in associations between taste sensitivity and weight status. Regression analyses were examined for influential cases and outliers (eg, studentized residuals, Cook’s distance, and centered leverage values) and multicollinearity (ie, variance inflation factor). Statistical significance was accepted as *P < *.05.

## RESULTS

### Study Characteristics

**Figure 1.**
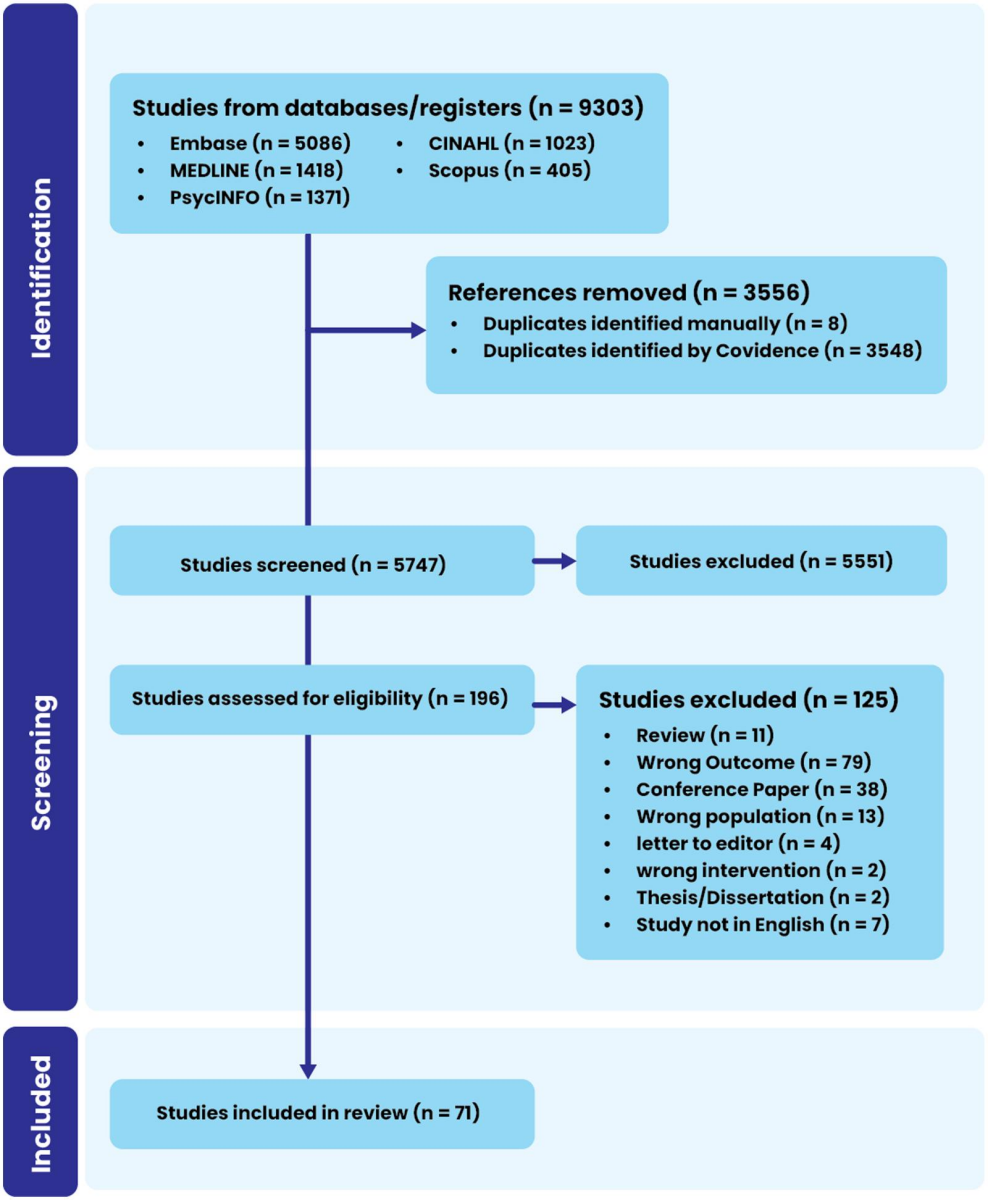
PRISMA (Preferred Reporting Items for Systematic reviews and Meta-Analyses) Diagram

In total, 9303 studies were identified using the systematic search method PRISMA (Figure 1). After duplicates were removed, 5741 records were screened by title and abstract, resulting in 196 articles being included for full-text review. Following the full-text review, 42 studies were included in the systematic review. Most studies (*n* = 41) employed a cross-sectional study design, with only 1 RCT.^42^ For included studies that assessed phenotype, most examined bitter taste sensitivity using STT methods (*n *= 22), with fewer employing RT (*n *= 5) and DT methods (*n *= 4). Additionally, 11 studies assessed bitter taste sensitivity using a genetic proxy (eg, TAS2Rs genotyping) without accompanying phenotypic measurements. Of the 42 studies, 27 had sufficient data to be included in the meta-analysis.^42–64^ Sample sizes ranged from small cohorts of 40 participants^65^ to larger-scale investigations with over 25,000 participants.^64^ Study quality assessment indicated 1 positive^42^ and 3 negative,^51^^,^^66^^,^^67^, with the remaining study having neutral results.

### Participant Characteristics

Study and participant characteristics are displayed in Tables  1and 2. Overall, 55% of included studies (*n* = 23/42) were conducted in Western nations with White populations (eg, United Kingdom, United States, Europe, Australia). The age range of participants varied across studies from 17^48^^,^^57^ to 96^68^ years of age. Most studies (*n* = 33/42) included male and female participants, with the remaining studies (*n* = 9/42) conducted only with female participants.^42–45^^,^^52^^,^^58^^,^^65^^,^^69^^,^^70^

**Table 1.** Study Characteristics of *n* = 27 Studies Included in the Meta-Analysis

Study No.	**1st Author**	Year	Country	Study type	Genotype	Taste testing	Age, yr	Female, %	Ethnicity majority	Sample Size	Weight status measurement	Study quality	Weight status measurement method
1	Inoue	2013	Japan	CS	TAS2R38	NR	18-22	100	Asian	87	Weight, BMI	Neutral	Professionally measured
2	Timpson	2005	United Kingdom	CS	TAS2R38	NR	60-79	100	Non-Hispanic White	3212	Weight, BMI, waist-hip ratio	Neutral	Professionally measured
3	Tepper	2002	United States	CS	NR	PROP	21-50	100	Non-Hispanic White	86	BMI	Neutral	Professionally measured
4	Deshaware	2017	India	CS	TAS2R38	PROP	19-55	46	Indian	393	BMI	Neutral	Professionally measured
5	Shen	2017	United Kingdom	CS	NR	PROP	18-55	70	White	136	BMI	Neutral	Professionally measured
6	Yackinous	2022	United States	CS	NR	PROP	17-36	62	Non-Hispanic White	114	Height, Weight, BMI	Neutral	Self-reported
7	Choi	2014b	Germany	CS	NR	PROP	18-55	56	Asian	350	BMI	Neutral	Professionally measured
8	Hoppu	2018	Finland	CS	TAS2R38	NR	NR	55	NR	1648	BMI, waist circumference	Neutral	Professionally measured
9	Ergun	2013	Turkey	CS	TAS2R38	PROP	NR	67	NR	178	Weight, BMI, waist-hip ratio, waist circumference, body fat percentage	Negative	Self-reported
10	Nagai	2017	Japan	CS	NR	PROP	18-26	100	Asian	153	Weight, BMI	Neutral	Self-reported
11	Keller	2013	Germany	CS	TAS2R38	NR	NR	60	Non-Hispanic White	1007	BMI, waist-hip ratio, body fat percentage	Neutral	Self-reported
12	Barajas-Ramirez	2016	Mexico	CS	TAS2R38	PROP	20-51	44	Hispanic	66	BMI, waist circumference	Neutral	Professionally measured
13	Wang	2022	China	CS	TAS2R38	PTC	18-23	58	NR	320	BMI	Neutral	Self-reported
14	Chupeerach	2021	Thailand	CS	TAS2R38	NR	18-35	79	Non-Hispanic White	657	BMI, body fat percentage	Neutral	Professionally measured
15	Trius-Soler	2022	Spain	CS	NR	PTC	17-29	NR	NR	390	BMI	Neutral	Professionally measured
16	Lambert	2019	United Kingdom	CS	TAS2R38	PTC	35-59	100	Non-Hispanic White	3328	BMI	Neutral	Self-reported
17	Villarino	2019	Philippine	CS	NR	PROP	18-60	82	Filipinos	100	BMI	Neutral	Professionally measured
18	Choi	2019	Korea	CS	TAS2R38	NR	40-89	63	Korean	3567	Weight, waist circumference, BMI	Neutral	Professionally measured
19	Khan	2020	Saudia Arabia	CS	NR	PTC	25-45	12	NR	200	BMI	Neutral	Professionally measured
20	Burgess	2017	United States	RCT	NR	PROP	18-60	100	White	107	BMI, Weight	Positive	Professionally measured
21	Yamaki	2023	Japan	CS	TAS2R38	NR	20-80	55	Japanese	2047	BMI	Neutral	Self-reported
22	Jo	2023	Korea	CS	TAS2R38	NR	40-69	49	Korean	3644	Weight, BMI, waist-hip ratio, waist circumference	Neutral	Professionally measured
23	Meng	2023	Canada	CS	TAS2R38	NR	45-85	50	NR	26 090	BMI, waist circumference	Neutral	Professionally measured
24	Choi	2014a	United States	CS	NR	PROP	NR	52	African American and Asian American	100	BMI	Neutral	Professionally measured
25	Goldstein	2005	United States	CS	NR	PROP	33-56	100	Non-Hispanic White	40	BMI, waist circumference, body fat percentage	Neutral	Professionally measured
26	Gupta	2018	India	CS	TAS2R38	PTC	14-22	42	Indian	303	BMI, waist-hip ratio, weight, waist circumference	Neutral	Professionally measured
27	Padiglia	2010	Italy	CT	NR	PROP	20-29	63	Non-Hispanic White	75	BMI	Neutral	Professionally measured

Abbreviations: BMI, body mass index; CS, cross-sectional; NR, not reported; PROP, 6-prophyltiuracil; PTC, phenylthiocarbamide; RCT, randomized control trial.

**Table 2.** Study Characteristics of *n* = 15 Studies Included in the Systematic Review Only

Study No.	1st Author	Year	Country	Study type	Genotype	Taste testing	Age, yr	Female, %	Ethnicity majority	Sample size	Weight status measurement	Study quality	Weight status measurement	Reason for exclusion from meta-analysis
1	Mikołajczyk-Stecyna	2017	Poland	CS	TAS2R38	NR	>60	100	Polish	118	BMI	Neutral	Professionally measured	No BMI/WHR data by genotype or diplotype.
2	Skrandies	2015	Germany	CS	NR	QHCL	19-56	62	German	66	BMI	Negative	Professionally measured	No BMI data by taster status.
3	Martinez-Cordero	2015	Mexico	CS	NR	Caffeine and QHCL	NR	46	NR	56	Weight, BMI	Neutral	Professionally measured	Data is only available by gender, not taster status.
4	Karmous	2018	Tunisia	CS	TAS2R38	PROP	NR	68	NR	104	Weight, BMI	Neutral	Professionally measured	Data only by weight category, not taster status.
5	Leon Bianchi	2018	Argentina	CS	NR	PROP	18-61	52	NR	66	BMI	Neutral	Self-reported	Data only by BMI category, not taster status.
6	Coletta	2013	United States	CS	NR	PROP, coffee, grapefruit juice	21-65	71	White	103	BMI	Neutral	Professionally measured	No detailed BMI data by taster group.
7	Drewnowski	2001	United States	CS	NR	PROP	18-70	52	White	751	BMI	Neutral	Self-reported	BMI data only by gender, not taster status.
8	Vignini	2019	United States	CS	NR	QHCL	NR	66	NR	71	BMI	Neutral	Professionally measured	No BMI data by taster status.
9	Gorovic	2011	Denmark	CS	TAS2R38	NR	50-64	50	White	500	BMI, waist circumference	Neutral	Self-reported	No BMI/WC data by taster status.
10	Turner	2021	Australia	CS	TAS2R4	NR	>65	55	NR	563	BMI, waist circumference	Neutral	Professionally measured	BMI data only by gender, not taster status.
11	Carta	2017	Italy	CS	NR	PROP	NR	69	White	110	BMI, waist circumference waist-hip ratio	Neutral	Professionally measured	Data only by weight category, not taster status.
12	Sharma	2013	India	CS	NR	PTC	20-50	100	Indian	105	Height, weight, BMI, body fat percentage	Negative	Professionally measured	Taster status is not categorized.
13	Cecati	2022	Italy	CS	TAS2R38 and TAS1R3	QHCL	NR	65	White	142	BMI	Neutral	Professionally measured	Data only by weight category, not taster status.
14	Fuchida	2013	Japan	CS	NR	QHCL	50-96	59	Sinhala	946	Height, weight, BMI	Neutral	Professionally measured	No BMI data by taster status.
15	Bahauddin	2023	Malaysia	CS	NR	PROP	20-45	70	NR	180	Weight, height, BMI	Neutral	Self-reported	Data only by weight category, not taster status.

Abbreviations: BMI, body mass index; CS, cross-sectional; PROP, 6-prophyltiuracil; PTC, phenylthiocarbamide; QHCL, quinine hydrochloride; WC, waist circumference.

### Bitter Taste Sensitivity and Anthropometric Measurement

Of the 42 studies included, bitter taste sensitivity was measured using phenotype in 22 studies,^42^^,^^45^^,^^47^^,^^49^^,^^52^^,^^57^^,^^59^^,^^61^^,^^65–68^^,^^71–80^ genotype in 12 studies,^43^^,^^44^^,^^50^^,^^53^^,^^56^^,^^60^^,^^62–64^^,^^69^^,^^81^^,^^82^ and both phenotype and genotype in 8 studies.^46^^,^^51^^,^^54^^,^^55^^,^^58^^,^^73^^,^^83^^,^^84^ Most studies that measured phenotype used PROP (*n* = 19/30) or PTC (*n* = 6/30).

All studies tested the *TAS2Rs* genes for genotype. All studies reported BMI measurements (*n* = 42), and some studies also reported measurements of waist–hip ratio (*n* = 6/42),^44^^,^^51^^,^^63^^,^^71^^,^^84^^,^^85^ waist circumference (*n* = 11/42),^50^^,^^51^^,^^54^^,^^60^^,^^63–65^^,^^71^^,^^81^^,^^82^^,^^84^ and body fat percentage (*n* = 5/42).^51^^,^^56^^,^^65^^,^^66^^,^^85^ In most studies (*n* = 31/42), the anthropometric measurements were undertaken by trained personnel, with the remainder using data from self-reported questionnaires (*n* = 11/42).^51^^,^^52^^,^^55^^,^^58^^,^^62^^,^^72^^,^^74^^,^^76^^,^^79^^,^^81^^,^^85^

### Association Between Bitter Taste Sensitivity and Weight Status

**Figure 2.**
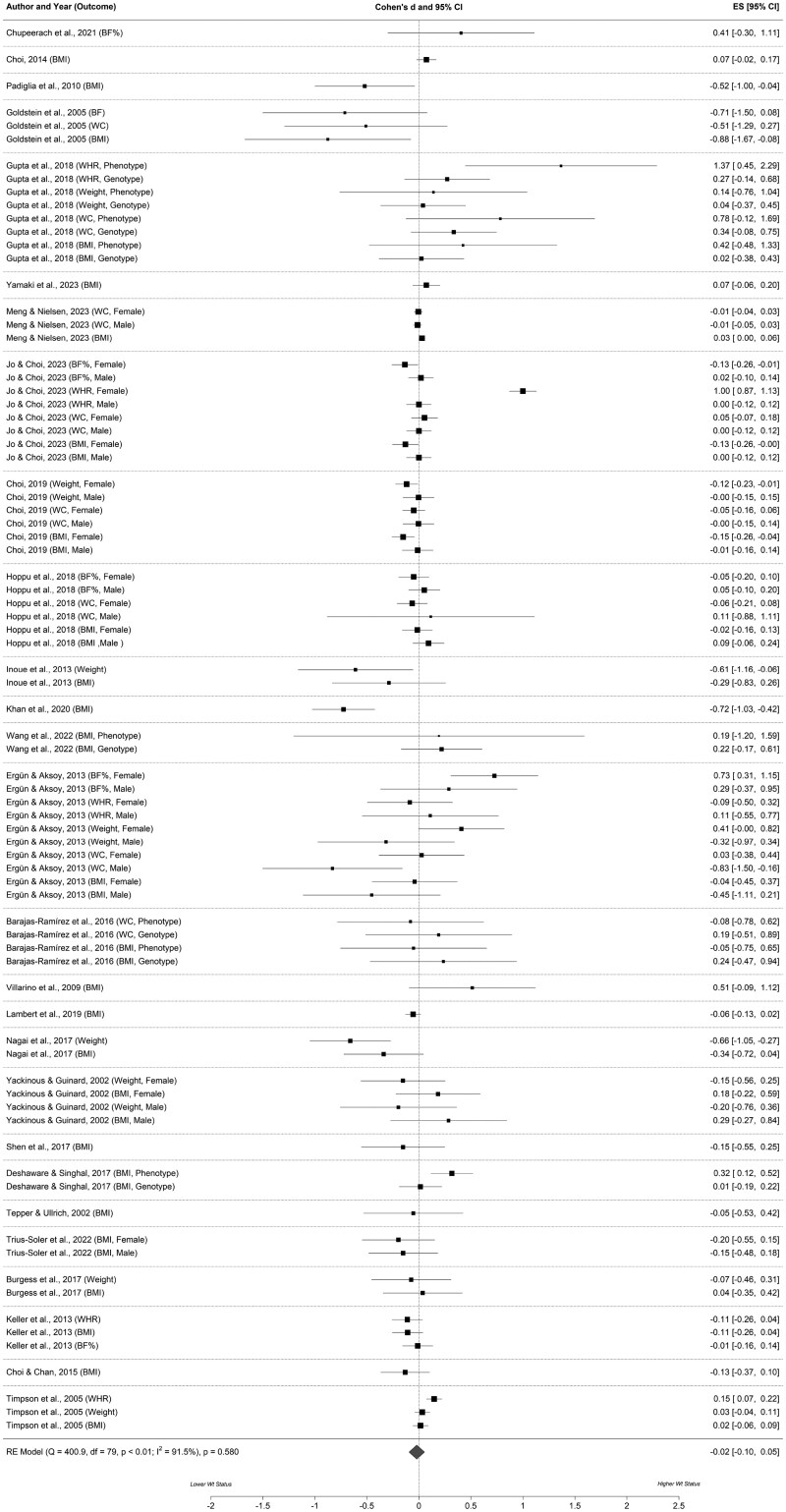
Forest Plot Examining 95 Multivariate Associations Between Bitter Taste Sensitivity and Weight Status

In total, 27 studies (*n* = 26 cross-sectional^43–47^^,^^49–65^^,^^78–80^^,^^84^; *n* = 1 RCT^42^) provided 80 effect estimates for the meta-analysis (Table 1). The weighted-mean effect (Figure 2) indicated no association between bitter taste sensitivity and weight status (Cohen’s *dz*, −0.02; 95% CI, −0.10 to 0.05; *P* = .58), with significant heterogeneity between studies (*Q* = 400.9, *df *= 79, *I*^2^=91.5%, *P < .*01). Outcomes from multilevel meta-regression that explored potential sources of heterogeneity and the influence of independent moderators are shown in Table S1. The lack of association between bitter taste sensitivity and weight status was consistent irrespective of study location, participant sex, outcome measurement, sample size, study design, and study quality. The type of outcome measured was identified as a significant independent moderator for weight status, with the association being notably higher (*P* = .004) for waist–hip ratio (coefficient for Cohen’s *dz*, 0.35) than weight (overall weight effect estimate was −0.02, 95% CI, −0.06 to 0.09). Visual inspection of the funnel plot suggested minimal publication bias (Figure S4), which was supported by a nonsignificant Egger’s test result (intercept, −0.0063; 95% CI, −0.13 to 0.12; *P* = * .*92). In our meta-analysis, most of the phenotype studies (*n* = 17) assessed bitter taste sensitivity using the ST intensity methods with PROP/PTC stimuli. Only 2 studies employed alternative methodologies: Khan et al.^61^ utilizd a DT approach, and Wang^55^ applied an RT method. Given the limited number of studies employing DT and RT methods, it was not feasible to conduct a subgroup analysis based on the assessed taste-sensitivity dimension.

### Subgroup Analysis

#### Nontasters vs Supertasters

The subgroup analysis between nontasters and supertasters included 18 studies, comprising 44 effect estimates. The weighted mean effect for weight status was nonsignificant (Cohen’s *dz*: −0.08, 95% CI, −0.20 to 0.04, *P = *.21). The associated forest plots are shown in Figure S1. Some asymmetry was observed in the funnel plot (Figure S5), hinting at possible publication bias. This was confirmed by a significant Egger’s test result (intercept: 0.08, 95% CI, −0.09 to 0.27, *P = *.03).

#### Nontasters vs Medium-Tasters

This comparison involved 17 studies and 42 effect estimates. The weighted mean effect for weight status was nonsignificant (Cohen’s *dz*,−0.02; 95% CI, −0.09 to 0.04; *P = *.43). The corresponding forest plots are shown in Figure S2. The funnel plot showed asymmetry, suggesting potential publication bias (Figure S6). However, this finding was not supported by the Egger’s test result (intercept, −0.01; 95% CI, −0.12 to 0.09; *P = *.80).

#### Supertasters vs Medium Tasters

Analysis of differences in weight status between supertasters and medium-tasters involved 17 studies, providing 42 effect estimates. The weighted mean effect for weight status was nonsignificant (Cohen’s *dz*, −0.05; 95% CI, −0.13 to 0.03; *P = *.26). Forest plots illustrating these results are included in Figure S3. The funnel plot appeared symmetrical, suggesting minimal visual evidence of publication bias (Figure S7). However, the Egger’s test indicated significant publication bias (intercept, 0.06; 95% CI, −0.05 to 0.18; *P = *.03).

### Studies Included Only in the Narrative Synthesis (ie, Systematic Review)

There are 15 cross-sectional studies included only in the systematic review aspect of this study (ie, sufficient data were not available to compute effect estimates to include in the meta-analysis). The details of the reason for exclusion can be found in Table 2. Of these studies, 3 reported a significant inverse association between bitter taste sensitivity and weight status,^76^^,^^83^^,^^86^ while 3 others reported a significant direct association.^68^^,^^71^^,^^82^ These studies were conducted between 2010 and 2021, with sample sizes ranging from 56 to 946 participants. Participants in the studies were aged 18 to 96 years and were predominantly (*n* = 4/6) of White populations (eg, European and Australian). Most studies measured phenotype (*n* = 4/6), while 2 studies measured genotype. Half of the studies measured BMI (*n* = 3/6) only, with the remainder also reporting other weight status outcomes.

Conversely, 9 studies found no significant association between bitter taste sensitivity and weight status.^66^^,^^67^^,^^69^^,^^72–75^^,^^77^^,^^81^ These studies were conducted between 2001 and 2023, with sample sizes ranging from 56 to 500 participants. Participants in these studies were aged between 18 and 64 years, and more than half of the studies (*n* = 6/9) were conducted within participants of White populations (eg, European and American). Most of these studies measured phenotype (*n* = 6/9), while 2 studies measured genotype,^69^^,^^81^ and 1 study measured both.^73^ Most of these studies measured BMI only (*n* = 5/9), with the remainder also reporting other weight status indicators.

## DISCUSSION

To the best of our knowledge, this study presents the first meta-analysis investigating the relationship between bitter taste sensitivity and weight status in the adult population. Overall, the pooled effect estimates indicate no significant relationship between bitter taste sensitivity and measures of weight status. This result suggests that differences in weight status that exist between individuals are unlikely to be related to differences in taste sensitivity categorization (ie, nontasters vs medium tasters vs supertasters).

While the meta-analysis revealed no significant association between bitter taste sensitivity and overall weight status, outcome type was identified in the meta-regression model as a significant independent moderator, with the outcome for waist–hip ratio significantly different to that of overall weight. This result aligns with previous research suggesting that taste perception could be linked more closely to fat distribution than overall body mass.^23^^,^^87^ However, this result should be interpreted cautiously for several reasons. First, the significant association for waist–hip ratio was derived from only 8 effect estimates across 5 studies.^44^^,^^51^^,^^53^^,^^63^^,^^84^ Methodological guidelines recommend a minimum of 10 studies for meta-regression, as smaller samples may lead to over- or under-estimations of the true effect size.^33^ Second, the quality of these 5 studies was generally low; with 4 rated as neutral^44^^,^^50^^,^^53^^,^^63^ and 1 as negative.^51^ Third, 1 of these studies exhibited poor between-group sample distribution, with 95 participants in the taster group compared to only 5 in the nontaster group, potentially skewing the results.^84^

In this systematic review, approximately 55% of the 42 included studies reported no significant association between bitter taste sensitivity and weight status. Notably, the study by Burgess et al.^42^ was the only RCT that included, focusing on the relationship between bitter taste sensitivity and weight status among American adults (*n* = 96). However, the data needed for meta-analysis were only available on baseline measurement, with no significant association observed between bitter taste sensitivity and either variable.^42^ This higher proportion of non-significant findings aligns with the overall non-significant result observed in our meta-analysis. The consistency between the lack of significant associations across individual studies and our aggregated meta-analytic outcome suggests that the non-significant relationship observed is likely to be a true reflection of the underlying association, rather than an artifact of selective reporting.

The absence of a significant association between bitter taste sensitivity and weight status was not only observed in the overall analysis but also within the subgroup analyses. Collectively, results indicate that the level of bitter taste sensitivity, whether between nontasters and supertasters, nontasters and medium-tasters, or medium-tasters and supertasters, does not significantly influence weight status. This finding may partly be attributed to methodological variability across the studies. Variations in study design, measurement techniques for taste sensitivity (eg, phenotypic or genotypic), weight status assessments (eg, self-reported vs clinically measured), and the outcomes measured (eg, weight, BMI, waist–hip ratio, waist circumference, and body fat percentage) may introduce inconsistencies in results. Additionally, bitter taste sensitivity has been evaluated using genotypic and phenotypic approaches, each employing distinct methodologies. For instance, genotypic assessments typically focus on the TAS2R38 receptor, utilizing various SNP and genotype classification systems like PAV/AVI^50^^,^^53–56^^,^^62^ CC/TT,^60^^,^^63^ or PV/AI.^43^ On the other hand, phenotypic measurements have been conducted using a range of methods, including PROP/PTC solutions,^42^^,^^45^^,^^46^^,^^51^^,^^52^^,^^54^^,^^59^^,^^65^^,^^78^^,^^83^^,^^84^^,^^88^ PTC strips,^61^ QHCL solutions,^67^^,^^68^^,^^73^^,^^77^^,^^86^ and bitter foods or beverages (eg, grapefruit juice, caffeine solutions).^75–77^ Among the compounds used to assess bitter taste sensitivity, PROP/PTC and quinine are most common; however, they differ in their receptor activation profiles.^89^^,^^90^ PROP/PTC is a highly specific agonist for the TAS2R38 receptor, whose common genetic polymorphisms (eg, PAV and AVI haplotypes) are strongly linked to individual differences in bitterness perception. This specificity has made PROP/PTC a reliable probe for studying TAS2R38-mediated sensitivity.^90^ In contrast, quinine is a more broadly tuned bitter compound that activates multiple TAS2Rs—up to 9 receptors in vitro—including TAS2R4, TAS2R7, TAS2R10, and TAS2R14.^91^ While this broader activation profile may better reflect the complexity of bitter perception in everyday foods, it also complicates receptor-specific interpretation and phenotype classification. These methodological differences in taste assessment may contribute to the variability seen in the literature and underscore the importance of clarifying the sensory and genetic basis of bitter taste in relation to weight-related outcomes.

In the current systematic review, only 8 studies utilized genotypic and phenotypic assessments.^46^^,^^51^^,^^54^^,^^55^^,^^58^^,^^73^^,^^83^^,^^84^ Combining these approaches is considered essential, as existing literature suggests that polymorphisms in TAS2Rs account for only 50%-80% of the variance in bitter taste sensitivity.^15^^,^^34^^,^^46^ It is recommended that future research employ a combination of subjective and objective methods for measuring bitter taste sensitivity to minimize self-reported bias. Additionally, employing trained personnel for weight status assessments rather than relying on self-reported data can also help minimize discrepancies and improve the accuracy of findings. Furthermore, the application of different statistical methods to control for confounding variables, including age, sex, and socioeconomic status, may also influence outcomes.^92^ These differences are crucial as they might affect the accuracy and comparability of measured outcomes across studies, potentially diluting associations that exist.^93^^,^^94^

Bitter taste sensitivity was the discrete variable under investigation in the present study; however, it is important to recognize that many other factors can influence weight status.^4^^,^^23^^,^^95–97^ Indeed, while genetic taste markers might predispose individuals to specific dietary preferences, actual food intake and anthropometric outcomes are also affected by environmental and behavioral factors.^98^ Another notable consideration is the interaction between genes and the environment, which is a significant factor in nutritional genomics.^99^ While certain genetic markers might predispose individuals to prefer certain tastes, actual consumption patterns are highly influenced by environmental factors such as food availability,^98^ socio-economic status,^92^and cultural dietary norms.^92^^,^^100^ For example, the availability of high-calorie, nutrient-poor foods in urban settings can have a greater impact on body weight and composition than genetic predispositions.^92^^,^^98^

### Strengths, Limitations, and Suggestions for Future Research

A major strength of this review is that it represents to our knowledge the first meta-analysis to systematically examine the relationship between bitter taste sensitivity and weight-related outcomes in adult populations. The review employed clearly defined eligibility criteria and rigorous analytical methods, synthesizing evidence across both phenotypic and genotypic measures. This comprehensive approach enhances relevance of the findings and provides a foundation for future research in this area. However, this review has some limitations.

The studies included in this meta-analysis primarily assessed TAS2R38-mediated sensitivity using PROP or PTC, as these were the most commonly studied receptor–stimulus combinations. While some research has examined other TAS2Rs or bitter compounds, we included these only in the narrative synthesis due to insufficient data for quantitative analysis, which suggests a valuable direction for future research to investigate a broader range of bitter taste receptors beyond TAS2R38 to enable more robust and comparable findings.

While our main objective was to examine the relationship between bitter taste sensitivity and weight status, we acknowledge that dietary intake and food preferences may act as confounding or mediating factors. However, most of the studies included in this review did not report or control for the variables of dietary intake and food preferences. As such, we were unable to incorporate this aspect of the topic as a moderating factor in our meta-regression analyses. Additionally, none of the included studies assessed trigeminal sensitivity or reported whether TAS2R38 agonists elicited irritation or pungency through co-activation of somatosensory pathways, such as TRPA1 receptors.^101^ As a result, we were unable to account for the potential confounding effects of these additional oral sensations in our analysis.

Although PROP and PTC are commonly used to assess TAS2R38 sensitivity under controlled conditions, this receptor also responds to several naturally occurring agonists found in foods, many of which co-occur with other bitter compounds and may engage multiple sensory systems concurrently. These more complex sensory interactions—beyond bitterness alone—were not captured in the available data and could potentially influence dietary behavior in ways not reflected by PROP/PTC-based assessments. Most studies included in the review (*n* = 41/42) were cross-sectional by design, limiting the potential to explore any causal relationships, although the associations in this review were not statistically significant. The potential for residual confounding remains, as these are observational studies. Nonetheless, we attempted to address the confounding factors most consistently reported in the included studies (eg, participant sex, study location, methods of measuring bitter taste sensitivity) by using adjusted models from the original investigations where possible.

Over half of the studies (*n* = 23/42) were conducted in Western countries with predominantly White participants. Our analysis is also limited to only peer-reviewed articles, which might lean toward publishing significant findings. Furthermore, this study only included articles published in English. Incorporating studies published in other languages could be advantageous for capturing a broader range of research, particularly involving non-White populations. Additionally, 2 subgroup analyses indicated potential publication bias based on Egger’s test, suggesting that findings from these analyses should be interpreted cautiously, as there may be an overrepresentation of studies with positive or significant results.

## CONCLUSION

This meta-analysis provides a comprehensive evaluation of the relationship between bitter taste sensitivity and weight status, showing no significant overall association across the included studies. However, our analysis highlights important nuances that warrant further investigation. While no clear link was found between bitter taste sensitivity and BMI or other anthropometric measures in the general population, the moderating effect of waist–hip ratio on this relationship suggests that the distribution of body fat may be influenced by taste sensitivity, even if overall weight or BMI is not. This finding underscores the complexity of factors driving obesity and suggests that future research can move beyond broad weight measures.

The heterogeneity across studies, driven by differences in methodologies, population demographics, and the way bitter taste sensitivity and weight status were assessed, further complicates the interpretation of results. Therefore, while this study offers valuable insights, it also highlights the need for more uniform research approaches and the importance of considering genetic, environmental, and behavioral factors in future investigations. Our findings suggest that the relationship between bitter taste sensitivity and weight status is multifactorial and influenced by a range of variables, including genetic polymorphisms, dietary patterns, and cultural practices.

To better understand the role of bitter taste perception in weight status, future research can aim for greater consistency in study design, consider the interaction between genetic and environmental factors, and expand the scope to include diverse populations. These suggestions will help build a more comprehensive framework that accounts for the complex interplay between taste perception and weight status, potentially informing more targeted public health strategies.

## Supplementary Material

nuaf160_Supplementary_Data
