# Supplementary material for: Association between Bitter Taste Sensitivity and Weight Status in Adults: A Systematic Review and Meta-Analysis
Source: Nutr Rev. 2025 Aug 28;84(1):84–98. doi: 10.1093/nutrit/nuaf160 (PMC12696387; doi:10.1093/nutrit/nuaf160)
Supplement: nuaf160_Supplementary_Data [file nuaf160_supplementary_data.docx]

**Supplementary Materials**

**Supplementary Table 1** Multi-level multivariate meta-regression to identify potential sources of heterogeneity when examining the association between bitter taste sensitivity and weight status

| **Study Characteristics** | **Coefficient (95% CI)** | ***p-value*** |
| --- | --- | --- |
| **Study location (WHO region)**  European region (reference group)  American region  Western Pacific region  Southeast Asia and Eastern Mediterranean region | F (3, 76) = 0.417, *p = 0.741*  0.004 (-0.210, 0.217)  0.012 (-0.189, 0.213)  0.014 (-0.121, 0.399) | 0.974  0.903  0.290 |
| **Sex**  Both boys and girls (reference group)  Girls  Males | F (2,77) = 0.713, P = 0.494  -0.089 (-0.256, 0.078)  -0.106 (-0.299, 0.085) | 0.290  0.272 |
| **Type of outcome measured**  Weight (reference group)  BMI  Waist circumference  Waist-hip ratio  Body fat percentage | F (4,75) = 2.723, P = 0.036  0.058 (-0.121, 0.237)  0.093 (-0.114, 0.299)  0.351 (0.115, 0.588)  0.146 (-0.084, 0.377) | 0.523  0.374  0.004  0.210 |
| **Outcome measurement**  Genotype (Tas2R38 gene)  Phenotype (PTC or PROP) | F (1, 78) = 0.03, P = 0.852  -0.084 (-0.223,0.057) | 0.240 |
| **Sample size**  <500 (reference group)  ≥500-<1000  ≥1000 | F (2, 77) = 0.415, P = 0.662  0.030 (-0.236, 0.296)  0.085 (-0.101, 0.272) | 0.825  0.365 |
| **Study design**  RCT/Cohort (reference)  Cross-sectional | F (1,78) = 0.061, P = 0.805  0.028 (0.120, 0.257) | 0.805 |
| **Study quality**  Positive  Neutral  Negative | F (2, 77) = 1.225, P = 0.299  0.268 (-0.092, 0.628)  0.331 (-0.130, 0.793) |  |

**Supplementary Table 2** Detailed PICO Frameworks

| Population | Generally healthy adult. Adults without chronic diseases, developmental disability, autism spectrum disorder or any other cognitive or intellectual impairment and sensory-based feeding difficulties (e.g., dysphagia). |
| --- | --- |
| Intervention | Any study design examining the association between bitter taste sensitivity (genotype and/or phenotype) and adult’s diet quality and weight status. |
| Comparison | Only applicable for study designs (e.g., Randomised Controlled Trials) with a comparative group i.e., tasters vs non-tasters. |
| Outcomes | Adult’s food intake, adult’s diet quality, adult’s weight status (BMI) |

**Supplementary Table 3** Systematic search strategy

| **Systematic search strategy based on PICO framework** | |
| --- | --- |
| 1 | exp ADULT/ or exp MEN/ or exp WOMEN/ or MALE/ or exp FEMALE/ |
| 2 | (adult* or male* or female* or men or women) |
| 3 | 1 or 2 |
| 4 | (bitter or “bitter taste” or taste* or “taste sensitivity*” or “taste threshold*) |
| 5 | (phenylthiocarbamide or PTC or 6-n-propylthiouracil or PROP or TAS2R38) |
| 6 | 4 or 5 |
| 7 | (adipos*) or exp obesity/ or exp overweight/ |
| 8 | (Underweight or “lower weight” or “undernourish*”) |
| 9 | (BMI or “Body Mass Index” or “weight status”) |
| 10 | (weight* or “waist circumference*” or “skinfold thickness” or “waist hip*” or Anthropometr*) |
| 11 | 7 or 8 or 9 or 10 |
| 12 | 3 and 6 and 11 |
| **The syntax used in this search strategy, Boolean operators (AND, OR and NOT), truncations (e.g., diet*) were adjusted where necessary according to the requirements of each database.* | |

**Inclusion and Exclusion Criteria**

Inclusions:

- As per the WHO’s definition of Adult, studies examining people aged 19 years old or older ^[1]^.
- Studies published in English with no restrictions on publication dates, type of study design, gender and study country.
- Reporting at least one quantitative analysis between bitter taste sensitivity (genotype and/or phenotype) with diet quality and/or weight status.
- Studies examining bitter taste sensitivity either as an independent or dependent variable.
- Studies with sufficient data to permit calculating effect estimates (e.g.,means) and associated variance (e.g.,95%CI) will be included in the meta-analysis.
- When multiple studies are published on the same participant cohort with same research questions, the publication with the largest sample size and/or more complete data set will be selected for the meta-analysis to prevent duplication^[2]^.

Exclusions:

- Studies with adults having chronic diseases, developmental disability, autism spectrum disorder or any other cognitive or intellectual impairment and sensory-based feeding difficulties (e.g. Dysphagia).
- Studies done exclusively on children, adolescents, or animals.
- Studies not published in English.
- Unpublished reports, dissertations, systematic/narrative/scoping reviews, letters-to-the-editors, editorials, commentaries, and case studies.

**Supplementary Table 4** Systematic search strategy based on the PICO framework.

| **PICO** | | **Inclusion Criteria** | **Exclusion Criteria** |
| --- | --- | --- | --- |
| Population | Adults without chronic diseases, developmental disability, autism spectrum disorder or any other cognitive or intellectual impairment and sensory-based feeding difficulties (e.g., dysphagia). | - Studies examining people aged 17 years old or older. | - Studies with adults having chronic diseases, developmental disability, autism spectrum disorder or any other cognitive or intellectual impairment and sensory-based feeding difficulties (e.g. Dysphagia). - Studies done exclusively on children, adolescents, or animals. |
| Intervention | Any study design examining the association between bitter taste sensitivity (genotype and/or phenotype) and adult’s diet quality and weight status. | - Studies reporting at least one quantitative analysis between bitter taste sensitivity (genotype and/or phenotype) with diet quality and/or weight status. - Studies examining bitter taste sensitivity either as an independent or dependent variable. - Studies with sufficient data to permit calculating effect estimates (e.g., means) and associated variance (e.g.,95%CI) will be included in the meta-analysis. - When multiple studies are published on the same participant cohort with same research questions, the publication with the largest sample size and/or more complete data set will be selected for the meta-analysis to prevent duplication^[2]^. - Studies published in English with no restrictions on publication dates, type of study design, gender and study country. | - Studies not published in English. - Unpublished reports, dissertations, systematic/narrative/scoping reviews, letters-to-the-editors, editorials, commentaries, and case studies. |
| Comparison | Only applicable for study designs (e.g., Randomised Controlled Trials) with a comparative group i.e., tasters vs non-tasters. | N/A | N/A |
| Outcomes | Adult’s food intake, adult’s diet quality, adult’s weight status (BMI) | N/A | N/A |

**Supplementary Table 5** Studies Assessing Phenotype Included in Meta-Analysis Based on Bitter Taste Dimensions

| **S. No** | **Author** | **Year** | **Study Design** | **Taste Sensitivity Dimension** | **Stimulus & Method** |
| --- | --- | --- | --- | --- | --- |
| 1 | Khan | 2020 | Cross Sectional | DT | PTC taste strip test, |
| 2 | Wang | 2022 | Cross Sectional | RT | PTC tasting ability threshold method; |
| 3 | Tepper | 2002 | Cross Sectional | STT | PROP; psychophysical classification |
| 4 | Deshaware | 2017 | Cross Sectional | STT | PROP solution method with LMS |
| 5 | Shen | 2017 | Cross Sectional | STT | PROP; LMS scale intensity ratings |
| 6 | Yackinous | 2022 | Cross Sectional | STT | PROP and NaCl; intensity comparisons and psychophysical categorisation |
| 7 | Choi | 2014b | Cross Sectional | STT | PROP paper disks; intensity rated using gLMS |
| 8 | Timpson | 2005 | Cross Sectional | STT | PTC tasting with gLMS |
| 9 | Ergun | 2013 | Cross Sectional | STT | PROP NaCl intensity comparisons with gLMS |
| 10 | Nagai | 2017 | Cross Sectional | STT | PROP (0.32 mM); intensity rated on LMS |
| 11 | Burgess | 2017 | RCT | STT | PROP; filter paper with 50 mM PROP rated on LMS |
| 12 | Barajas-Ramirez | 2016 | Cross Sectional | STT | PROP intensity scaling using LMS |
| 13 | Choi | 2014a | Cross Sectional | STT | PROP filter paper disk (50 mM); gLMS used for intensity rating |
| 14 | Goldstein | 2005 | Cross Sectional | STT | PROP filter paper disks (50 mM); LMS intensity ratings |
| 15 | Trius-Soler | 2022 | Cross Sectional | STT | PTC solutions with LMS |
| 16 | Lambert | 2019 | Cross Sectional | STT | PTC tasting with LMS |
| 17 | Villarino | 2019 | Cross Sectional | STT | PROP solutions (0.032, 0.32, 3.2 mM); LMS scale |
| 18 | Gupta | 2018 | Cross Sectional | STT | PTC solution method |
| 19 | Padiglia | 2010 | Cross Sectional | STT | PROP solutions (0.032, 0.32, 3.2 mmol/L) with gLMS |

Abbreviations:

DT : Detection Threshold

RT : Recognition Threshold

STT : Suprathreshold Taste Intensity

LMS : Labelled Magnitude Scale

gLMS: General Labelled Magnitude Scale

RCT: Randomised Controlled Trial

**Supplementary Table 6** List of IVs and DVs in Each Study and Their Controlled Variables

| **Study Number** | **Author, year** | **Independent Variables** | **Dependent Variables** | **Controlled Variables** |
| --- | --- | --- | --- | --- |
| 1 | Inoue et al. (2013) | TAS2R38 genotype (PROP taster status) | Height, Weight, BMI, Energy, and Nutrient Intake | None |
| 2 | Timpson et al. (2005) | TAS2R38 haplotypes | CHD, BMI, Diabetes, Eating behaviour | Age |
| 3 | Tepper & Ullrich (2002) | PROP taster status | BMI | Dietary restraint, Disinhibition |
| 4 | Deshaware & Singhal (2017) | TAS2R38 genotype, PROP taster status | BMI, Food preferences | None |
| 5 | Shen et al. (2016) | PROP taster status, CD36 and CA6 genotypes, FPD | Liking of ice cream, Dietary fat intake | Age, Gender, Ethnicity, and Salt taste sensitivity. |
| 6 | Yackinous & Guinard (2002) | PROP taster status | Dietary intake, Energy from fat, Fruit/vegetable consumption, BMI | Fungiform papillae count, Gender |
| 7 | Choi & Chan (2014) | PROP taster status, Chili pepper usage | BMI, Energy intake, Fat intake | Age, Sex, Ethnicity |
| 8 | Hoppu et al. (2018) | TAS2R38 genotype | BMI, Waist Circumference, Body Fat % | Sex, Age |
| 9 | Ergun et al. (2013) | TAS2R38 genotype | Food choices, BMI, Body fat % | Gender |
| 10 | Nagai et al. (2017) | PROP taster status | Food preferences (esp. vegetables), BMI, Height, Weight | Age |
| 11 | Keller et al. (2013) | TAS2R38 genotype (PAV/AVI, etc.) | Body fat %, Glucose levels, Smoking, Alcohol intake | Sex |
| 12 | Barajas-RamÃ­rez et al. (2016) | TAS2R38 genotype, PROP taster status | Taste sensitivity (PROP, capsaicin, linoleic acid), Food intake, BMI | Dietary restraint (TFEQ) |
| 13 | Wang et al. (2022) | TAS2R38 genotype, PTC sensitivity | BMI, Food preference, Health status | Sex |
| 14 | Chupeerach et al. (2021) | TAS2R38 polymorphisms (PROP taster genotype) | Body fat %, Obesity risk | Race/ethnicity, Age, Gender |
| 15 | Trius-Soler et al. (2022) | PTC taster status | BMI, Taste recognition thresholds, Bitter vegetable intake | Sex |
| 16 | Lambert et al. (2019) | PTC taster status, TAS2R38 diplotype | Cancer risk, Bitter food intake | Age, Socioeconomic status, Smoking status |
| 17 | Villarino et al. (2009) | PROP taster status | Food preferences (sweet, bitter, meat, beverages), BMI | Age, Sex |
| 18 | Choi (2019) | TAS2R38 rs10246939 genotype | BMI, Body weight, Food intake (fruits, cruciferous vegetables) | Sex, Age, Smoking, Alcohol consumption, Exercise, Education |
| 19 | Khan et al. (2020) | PTC taster status, Fungiform papillae count | BMI | Smoking status, Gender |
| 20 | Burgess et al. (2017) | PROP taster status (NT vs. ST), Diet type (Low-Carb vs. Low-Fat) | Weight loss | PROP status |
| 21 | Yamaki et al. (2023) | TAS2R38 genotype | Cruciferous vegetable intake, Alcohol intake, BMI | None |
| 22 | Jo & Choi (2023) | TAS2R38 rs10246939 genotype | BMI, Body Fat %, Obesity degree | Sex |
| 23 | Meng & Nielsen (2023) | TAS2R38 haplotypes (PAV/AVI) | Vegetable, Sweet food intake, Alcohol consumption, HDL, VAI | Age, Sex, Lifestyle, Socioeconomic factors |
| 24 | Choi (2014a) | PROP taste intensity, Race (African vs. Asian Americans) | BMI, Food liking (fatty, sweet foods) | Sex, Age, Food liking scores |
| 25 | Turner et al. (2021) | TAS2R4 and TAS2R5 polymorphisms | BMI | Sex |
| 26 | Gupta et al. (2018) | TAS2R38 genotype (A49P), PTC sensitivity | Waist circumference, Waist-hip ratio, BMI | Sex |
| 27 | Padiglia et al. (2010) | PROP taster status, CA6 gene polymorphism (rs2274333), Salivary zinc | BMI | Taste thresholds, Eating behaviour (TFEQ), Zinc levels |
| 28 | MikoÅlajczyk-Stecyna et al. (2017) | TAS2R38 and CA6 genotypes | Bitter food intake, BMI, Glucose, CRP, Lipid profile | Age |
| 29 | Skrandies & Zschieschang (2015) | BMI categories | Taste and smell thresholds (salty, sweet, olfactory) | None |
| 30 | Martinez-Cordero et al. (2015) | Taste thresholds (sweet, salty, sour, bitter) | BMI, Energy intake | Sex, hormone levels (leptin) |
| 31 | Karmous et al. (2017) | PROP sensitivity, CD36 and TAS2R38 SNPs | Fat and bitter taste thresholds, BMI | BMI classification |
| 32 | Leon Bianchi et al. (2018) | PROP concentration, Bitter beverage types | Bitterness intensity, Hedonic ratings, Reaction time (R-T), BMI | BMI classification |
| 33 | Coletta et al. (2013) | PROP taster status (Super-tasters vs. Non-tasters) | Change in energy intake, BMI | Baseline dietary intake (energy, macronutrients, food groups) |
| 34 | Drewnowski et al. (2001) | PROP taster status (phenotypic via filter paper) | Bitterness perception, Sweetness ratings (aspartame), BMI | Age, Sex |
| 35 | Vignini et al. (2019) | Taste stimulus identification ability (6 tastants including fat) | Taste sensitivity score, BMI | Age, Gender |
| 36 | Gorovic et al. (2011) | TAS2R38 genotypes | Brassica vegetable intake, BMI, Waist circumference, Dietary habits | None |
| 37 | Carta et al. (2017) | PROP taster status (NT, MT, ST) | BMI, Plasma endocannabinoids, Lipid metabolism markers | BMI classification |
| 38 | Turner et al. (2021) | TAS2R4, TAS2R5 genotypes | BMI | Sex |
| 39 | Sharma et al. (2013) | PTC taster status (TAS2R38 phenotype) | PMS severity, Body fat, BMI | Age, Menstrual regularity, Adiposity |
| 40 | Cecati et al. (2022) | TAS2R38 and TAS1R3 polymorphisms | Sweet taste recognition, Overall taste sensitivity, BMI | Age, Sex, Genetic variants |
| 41 | Fuchida et al. (2013) | Bitter taste sensitivity (QHCL threshold) | Underweight status (BMI < 18.5) | Age, Sex, and Medication use. |
| 42 | Bahauddin et al. (2023) | PROP taster status | Sweet food intake, Fruit consumption, Total dietary intake | BMI, Age, Sex |

**Supplementary Table 7** Overview of Variables and Study Counts for Meta-Analysis

| **Variables** | **Measurement** | **Number of Studies for Meta-Analysis** |
| --- | --- | --- |
| Bitter Taste Dimension  (Phenotype Assessment) | Suprathreshold Method  Detection Method  Recognition Method | 17  1  1 |
| TAS2Rs Genotype | TAS2R38  Other TAS2Rs Gene | 15  0 |
| Bitter Taste Sensitivity Status | Taster (Medium, Super) VS Non-Taster  Other Method | 27  0 |

**
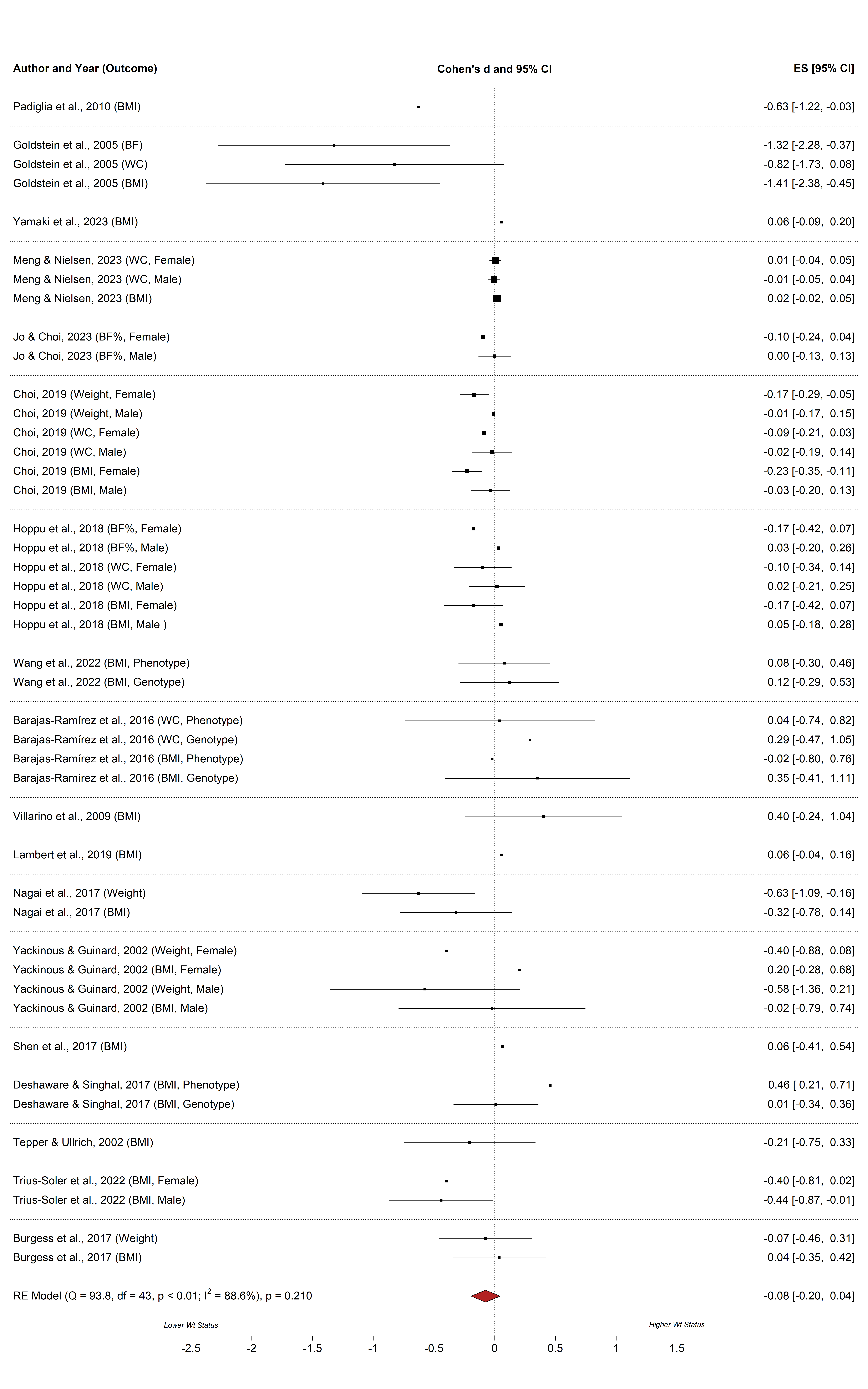
**

**Supplementary Figure 1** Forest plot examining 44 multivariate associations between non-taster and super-taster groups with weight status

**
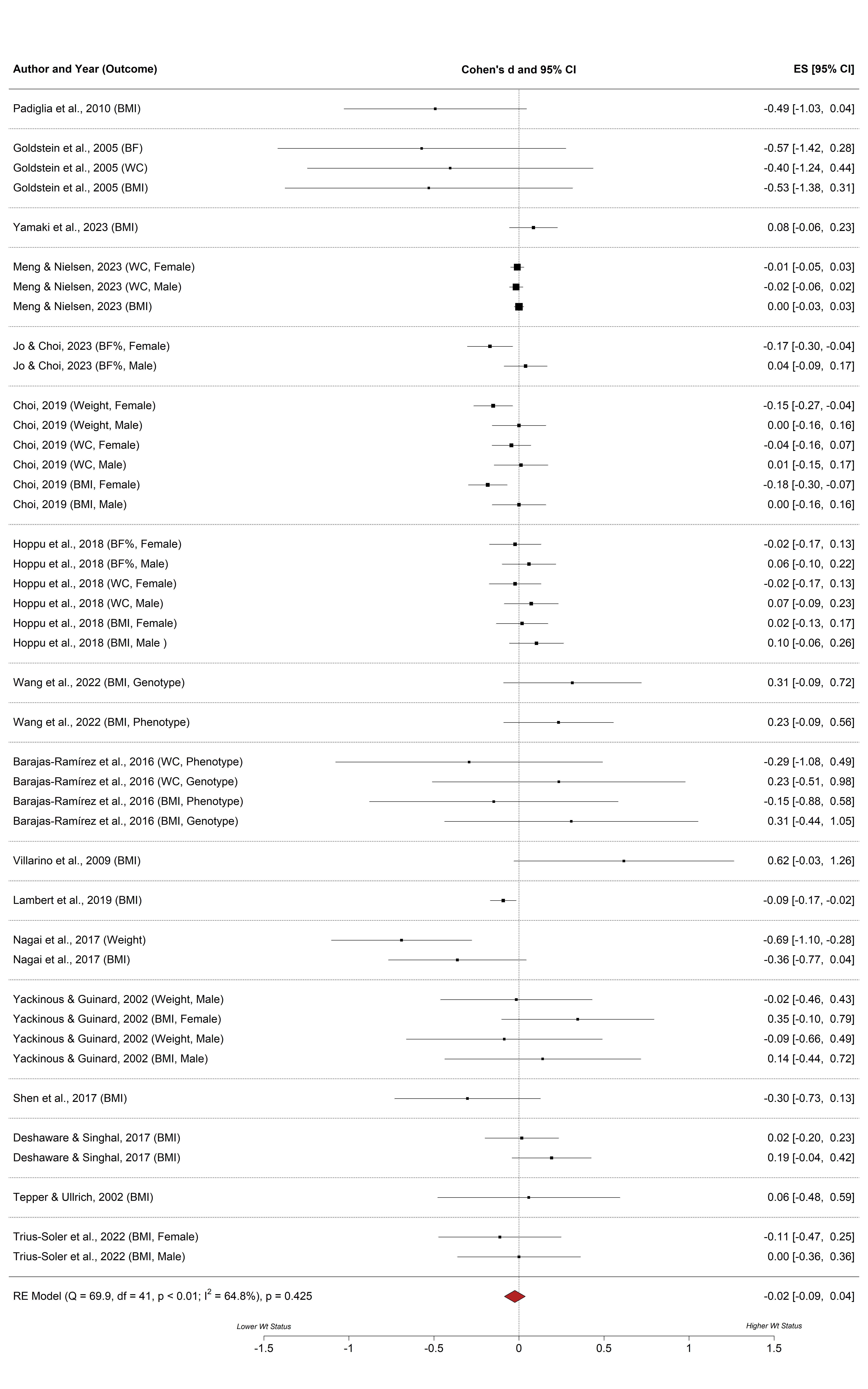
**

***Supplementary Figure 2.*** *Forest plot examining 42 multivariate associations between non-taster and medium-taster groups with weight status*

**
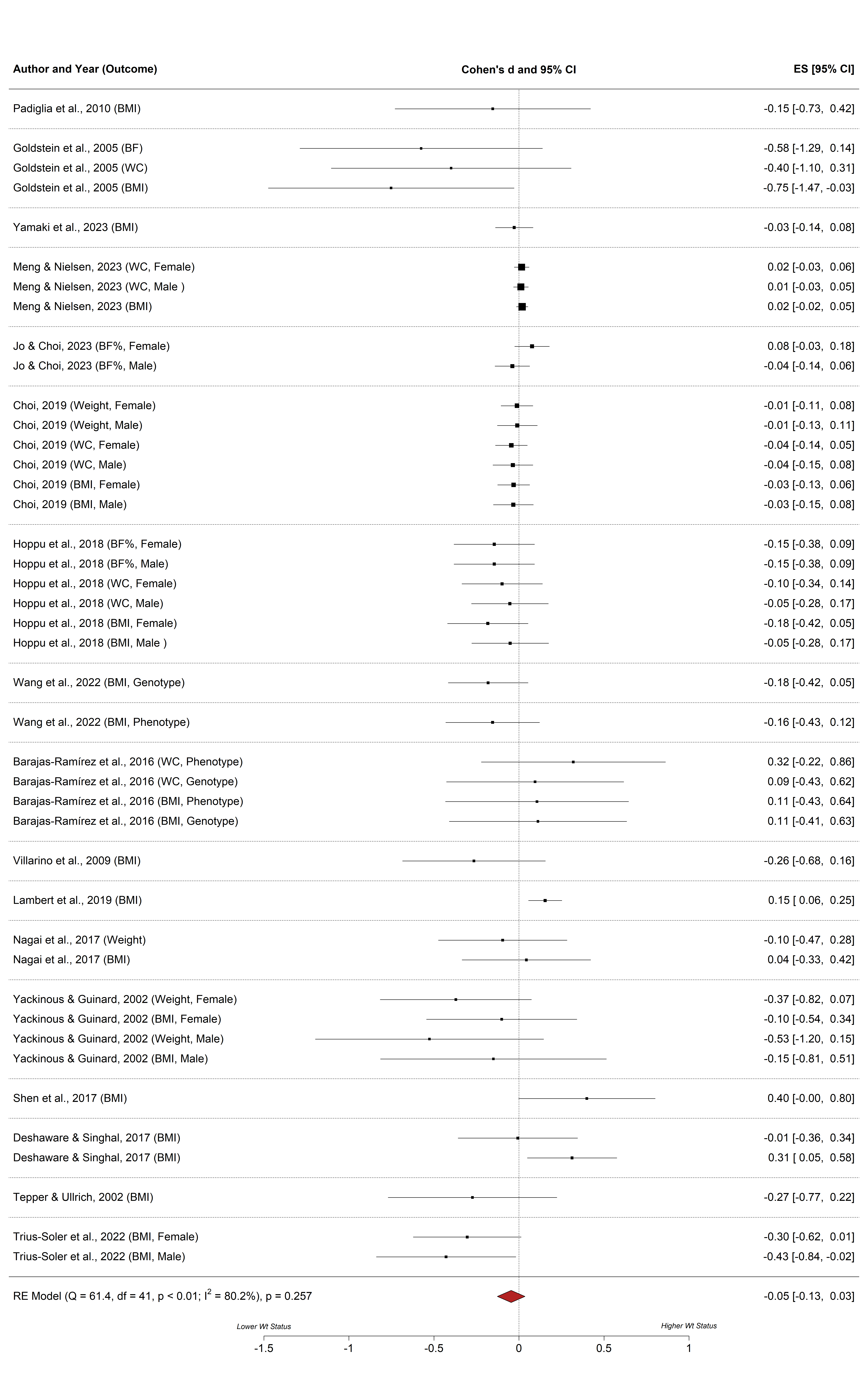
**

**Supplementary Figure 3** Forest plot examining 42 multivariate associations between medium-taster and super-taster groups with weight status

**
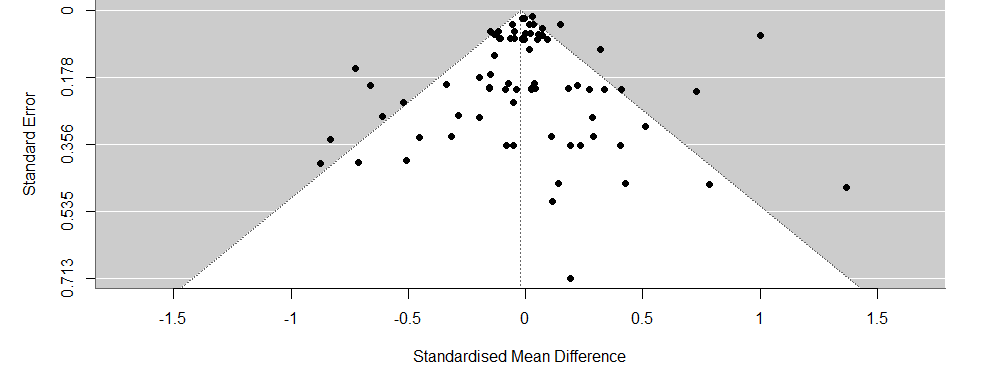
**

**Supplementary Figure 4** Assessment of publication bias-Funnel plot: bitter taste sensitivity and weight status (overall)

**
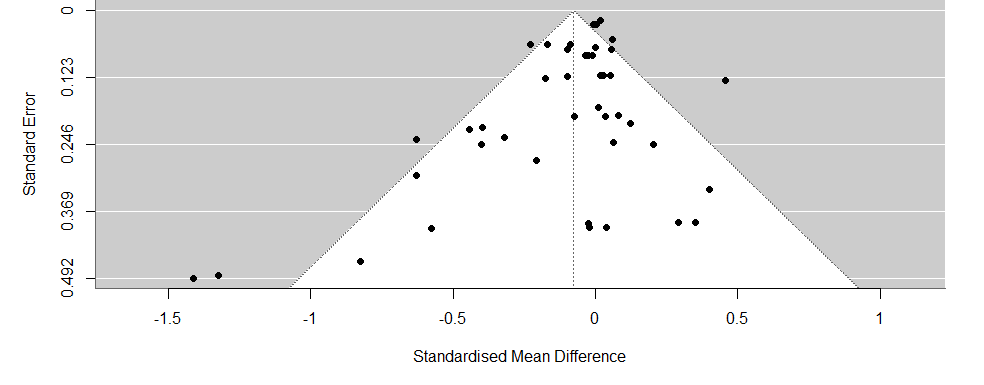
**

**Supplementary Figure 5** Assessment of publication bias-Funnel plot: non-taster and super-taster

**
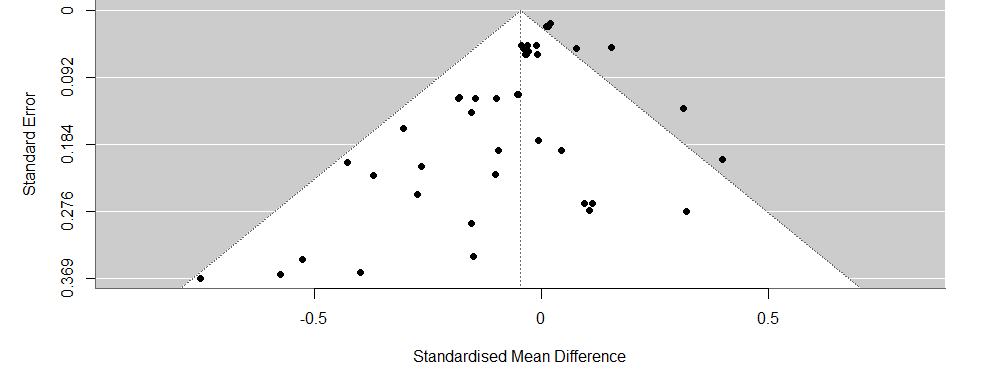
**

**Supplementary Figure 6** Assessment of publication bias-Funnel plot: non-taster and medium-taster

**
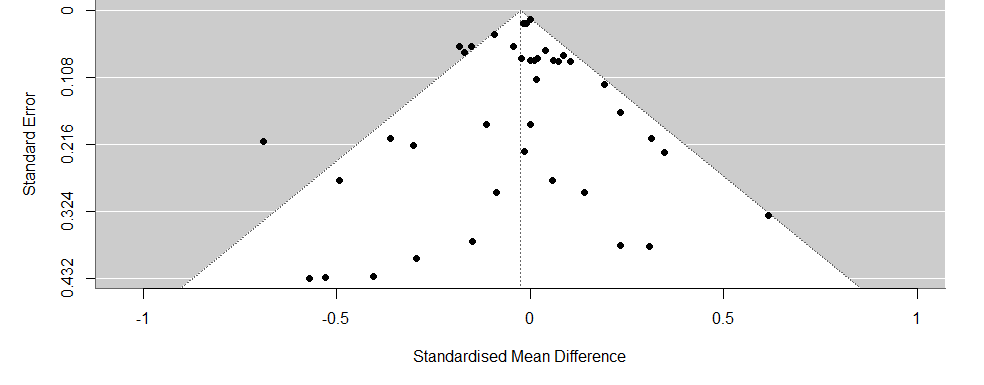
**

**Supplementary Figure 7** Assessment of publication bias-Funnel plot: medium taster and super taster
